# Polymer-encased nanodiscs with improved buffer compatibility

**DOI:** 10.1038/s41598-017-07110-1

**Published:** 2017-08-07

**Authors:** Mariana C. Fiori, Yunjiang Jiang, Guillermo A. Altenberg, Hongjun Liang

**Affiliations:** Department of Cell Physiology and Molecular Biophysics, and Center for Membrane Protein Research, Texas Tech University Health Sciences Center, Lubbock, Texas USA

## Abstract

Styrene-maleic acid copolymers allow for solubilization and reconstitution of membrane proteins into nanodiscs. These polymer-encased nanodiscs are promising platforms for studies of membrane proteins in a near-physiologic environment without the use of detergents. However, current styrene-maleic acid copolymers display severe limitations in terms of buffer compatibility and ensued flexibility for various applications. Here, we present a new family of styrene-maleic acid copolymers that do not aggregate at low pH or in the presence of polyvalent cations, and can be used to solubilize membrane proteins and produce nanodiscs of controlled sizes.

## Introduction

Approximately 30% of genes in sequenced genomes correspond to membrane proteins (MPs), which are the targets of most drugs in medical use^1, 2^. Accordingly, there is great interest in understanding the structure and function of this class of proteins. Integral MPs are harder to study than soluble proteins because they require a heterogeneous environment compatible with their hydrophobic and hydrophilic regions that normally interact with membrane lipids and the aqueous solutions on both sides of the membrane, respectively. For many studies, MPs are extracted from cell membranes with detergents, purified when still solubilized in detergent micelles, and then assayed in detergent micelles, in solubilized forms with other amphipathic molecules, or after reconstitution into lipid membranes. The use of detergents is relatively simple, but detergent micelles are far from ideal as MP-supporting platforms. Differences in physicochemical properties such as curvature, lateral pressure profile and thickness, and dynamic equilibrium of free detergent molecules in solution and that in the micelles, contribute to the reduced stability of MPs compared to that in biomembranes^3–5^. Moreover, there are reports of significant structural and/or functional differences between MPs studied in detergent micelles and in lipid bilayer membranes^6–11^.

Reconstitution of MPs into liposomes increases their stability, but significant drawbacks include the limited accessibility to the intraliposomal side and the undesirable light scattering caused by the relatively large sizes of liposomes, which complicates optical spectroscopy measurements. Nanodiscs (NDs) are a desirable alternative that consists of two copies of a membrane scaffold protein that encase a small lipid bilayer patch^11^. Under optimal reconstitution conditions, NDs constitute a homogeneous and monodisperse population of proteolipid nanostructures that can be treated as soluble proteins. This is particularly advantageous for the implementation of methodologies such as fluorescence and solution NMR spectroscopies and single-particle electron cryo-microscopy^10, 11^. A recent variant of the NDs are the polymer-encased NDs also known as styrene-maleic acid (SMA)-lipid particles (SMALPs) or lipodisqs. In SMALPs the membrane scaffold proteins are replaced by amphipathic SMA copolymers^12–14^. There are a few features of SMALPs that make them highly desirable. For example, SMA copolymers can be used to solubilize native membranes directly, maintaining a close to physiologic environment for MPs, without ever using the often disruptive detergent solubilization process^12, 14^. Also, SMALPs can be produced from membranes of well-controlled lipid compositions, allowing studies of the role of membrane lipids on regulating MP functions^12–14^. Additionally, SMA copolymers do not exhibit superimposed adsorption peaks with MPs, which is a significant advantage for far-UV circular dichroism spectroscopy studies of MPs^15^.

In spite of the current excitement about SMALPs, their use is limited by the incompatibility of SMA with low pH solutions and polycationic solutes. With pK_a_s of ~6 and ~10 of the two carboxyl groups of each maleic acid repeating unit^16^, SMA becomes neutral at low pH and precipitates out from solution due to hydrophobic interactions. The presence of carboxyl groups can also interfere with the purification and functional studies of many MPs because of the coordination of the SMA carboxyl acids with the immobilized Ni^2+^ or Co^2+^ used for His tag-based purification, and SMA precipitation that follows electrostatic association of the carboxyl acids with Ca^2+^, Mg^2+^ and polyvalent cations in general^12, 17^. As a result, assays that require lowering pH to 6 or less (*e.g*., determination of the spectral shifts of rhodopsins in response to acidification, activation of ion channels such as KcsA) or studies of ATPases that require Mg^2+^ (*e.g*., ion pumps, ATP-binding cassette proteins) cannot be performed with the MPs reconstituted in SMALPs. Here, we addressed the known buffer incompatibilities of SMALPs through the synthesis of a new family of SMA copolymers (zSMA; z for zwitterionic) that can be used to solubilize membrane proteins into NDs of controlled size.

## Results

### New cysteamine-PC modified alternating polymer P(S-*at*-MA)

**Figure 1:**
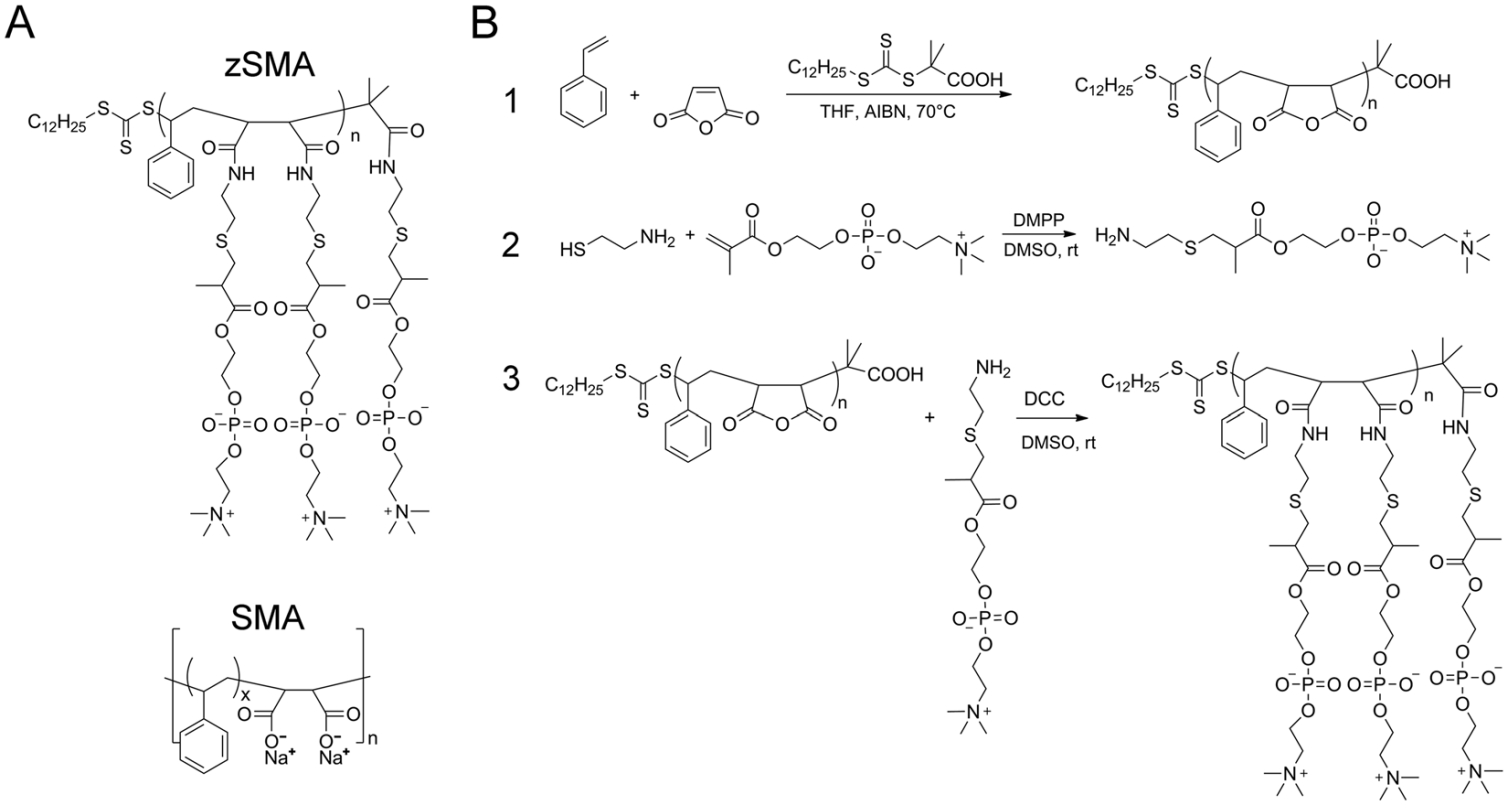
New family of zSMA copolymers. (**A**) Structures of the zSMA copolymers prepared by RAFT polymerization (top) compared with conventional SMA (bottom). The letter n signifies the number of repeating units. In the most frequently used commercial copolymers x ~3. (**B**) Reaction scheme for the synthesis of zSMAs.

To address the buffer incompatibilities of SMA we designed and synthesized a new family of SMA copolymers where the carboxyl acids were replaced with zwitterionic phosphatidylcholine (PC) groups (Fig. 1A), hereafter referred as zSMA. Even though maleic acid is not part of the new copolymers, we decided to name them zSMAs (z for zwitterionic) because of the widespread use of the term SMALPs. We synthesized three new zSMA copolymers where the number of repeat units (n in Fig. 1A) was 59 (zSMA1), 106 (zSMA2), and 215 (zSMA3), respectively. The alternating copolymers P(S-*at*-MA) of different size were synthesized by the reversible addition-fragmentation chain-transfer (RAFT) polymerization method with *S*-1-dodecyl-S′-(α,α′-dimethyl-α″-acetic acid)trithiocarbonate (DATC) as the chain transfer agent and azobisisobutyronitrile (AIBN) as the initiator (reaction schemes are presented in Fig. 1B).

**Figure 2:**
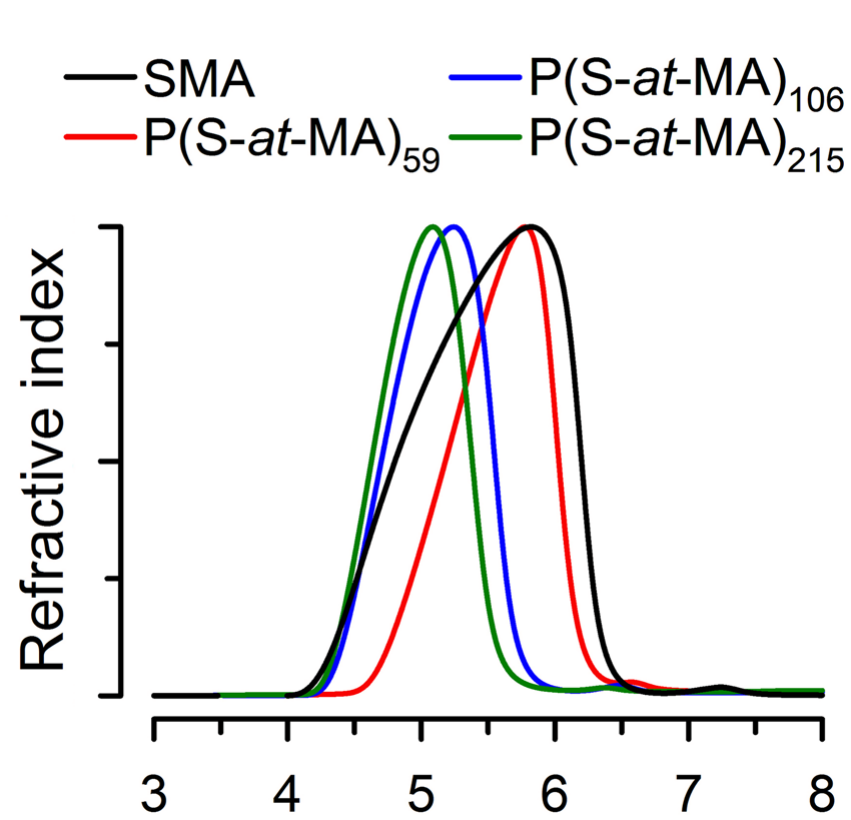
SEC of P(S-*at*-MA) alternating copolymers and SMA. SMA (Xiran), P(S-*at*-MA)_59_, P(S-*at*-MA)_106_ and P(S-*at*-MA)_215_ were run on an Agilent PLgel 5 µm MIXED-D column equilibrated with DMF/0.02 M ammonium acetate, and run at 0.5 ml/min and 50 °C. Elution was monitored by following the refractive index. Each trace was normalized to its peak.

The analysis of the P(S-*at*-MA)s by size-exclusion chromatography (SEC) (Fig. 2) showed a pattern of elution consistent with the size sequence expected from the molecular weights determined from conversion and NMR analysis (Supplementary Tables); zSMA1: 12,451/12,675 (conversion/NMR), zSMA2: 21,576/21,777, and zSMA3: 43,708/43,795. For SEC analysis, the light scattering signals in our system were often too weak to determine molecular weight accurately (Supplementary Table 2), although we could calculate a polydispersity index (PDI) of 1.085 for zSMA1, 1.170 for zSMA2 and 1.197 for zSMA3. These values are smaller than that calculated for the SMA control (1.341). Overall, these data indicate that our zSMAs have well-defined sizes.

### Production of (zSMA)-lipid particles

**Figure 3:**
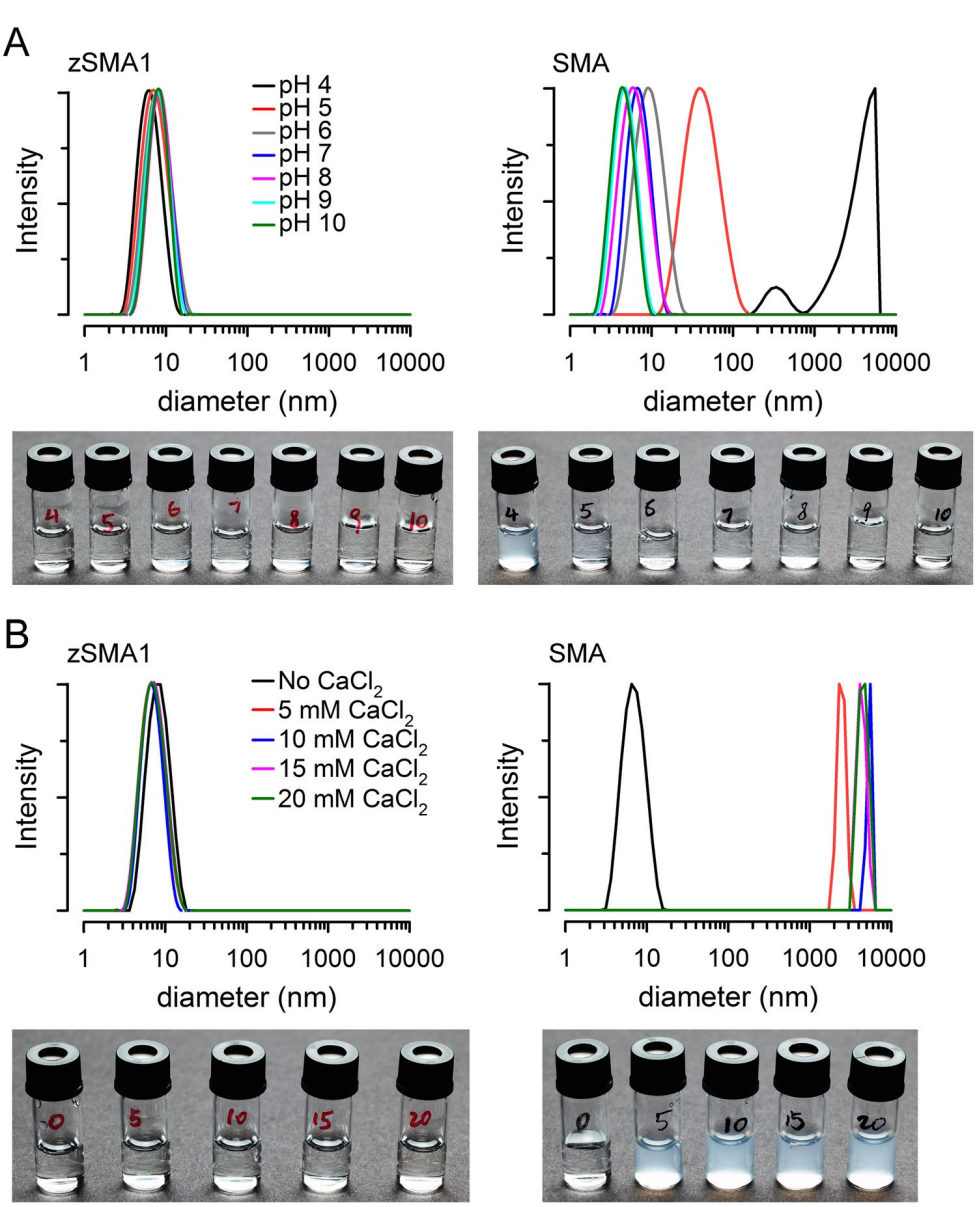
A comparison of buffer compatibility between zSMA1 copolymer and SMA. (**A**) Effect of pH on a solution containing 1 mg/ml SMA (Xiran) or zSMA1. We used 50 mM acetate/sodium acetate as buffer for pHs 4 and 5, 50 mM K_2_HPO_4_/KH_2_PO_4_ for pHs 6 and 7, 50 mM Tris/HCl for pHs 8 and 9, and 50 mM Ches/NaOH for pH 10. Aggregation was followed by the changes in apparent diameter determined by DLS, and were also assessed by direct visualization (pictures). (**B**) Effects of addition of CaCl_2_ on aggregation. CaCl_2_ was added to the copolymers dissolved in water to reach the concentrations indicated. See legend of panel A for details.

Some of the incompatibilities of SMA are illustrated in Fig. 3. Lowering pH (Fig. 3A, right) or addition of CaCl_2_ (Fig. 3B, right) results in aggregation and precipitation of Xiran SL25010-S25 (a gift from Polyscope Polymers) as an example of SMA copolymers. This compound, simply referred as SMA in the text, is a styrene-maleic acid copolymer with a molar ratio of styrene-to-maleic acid of 3:1 and an average molecular mass of 10 kDa^18–21^. In contrast, as expected from its design without the carboxyl acids, lowering pH (Fig. 3A, left) or adding CaCl_2_ (Fig. 3B, left) does not produce aggregation and precipitation of zSMA1.

**Figure 4:**
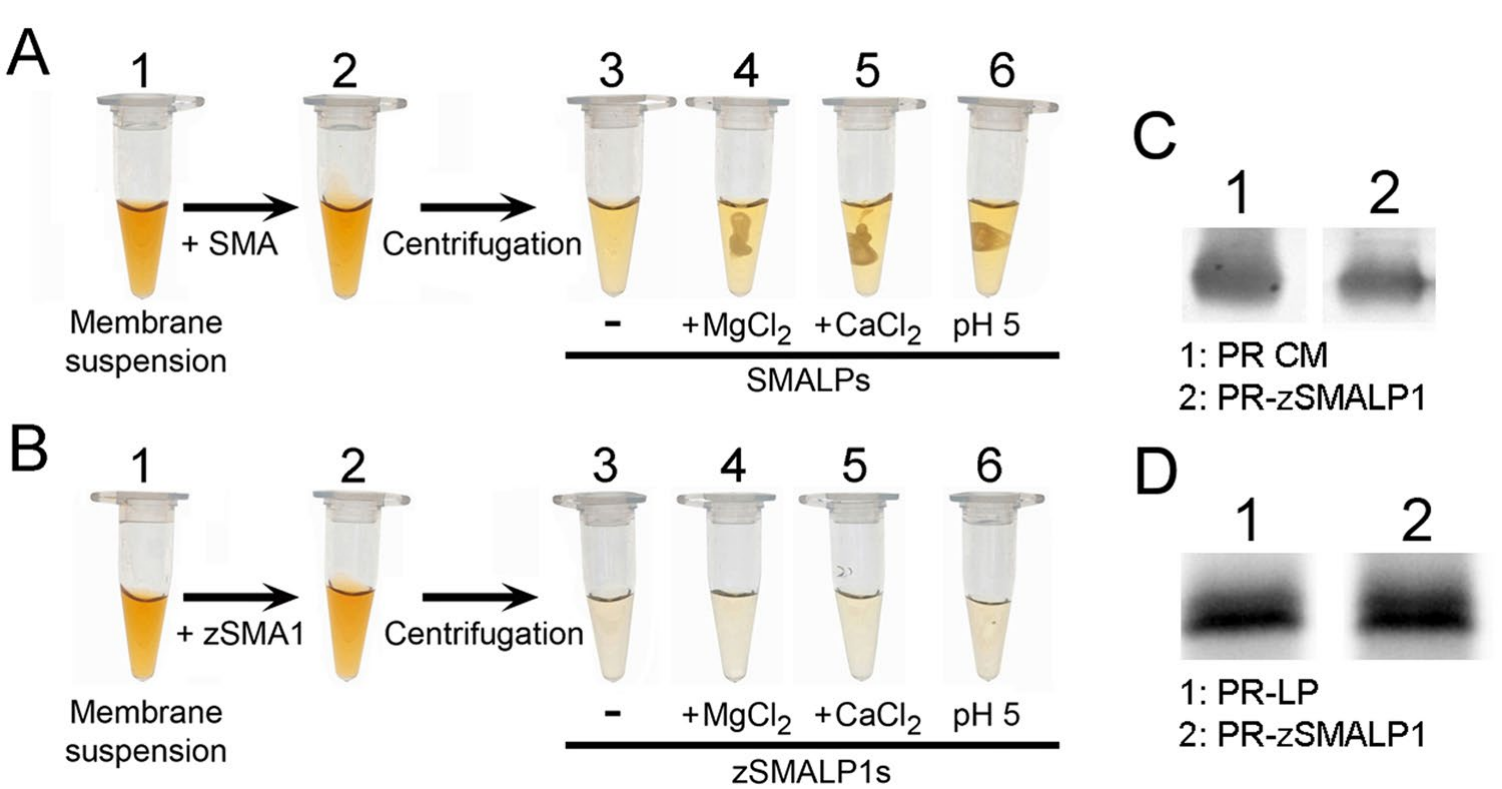
A comparison of buffer compatibility between zSMALP1s and SMALPs. (**A**) SMA nanodiscs (SMALPs). Crude membranes from *E. coli* expressing recombinant PR (tube 1) were solubilized with SMA (Xiran SL25010-S25; tube 2). The solubilized material obtained after centrifugation was visualized in the absence of buffer changes (tube 3) or after addition of MgCl_2_ (tube 4), CaCl_2_ (tube 5) or HCl (tube 6). The final concentrations of MgCl_2_ and CaCl_2_ were 5 mM. The pH in all tubes was 8, except for tube 6 were pH was 5. (**B**) zSMA1 nanodiscs (zSMALP1s). See legend of panel A for details. (**C**) Solubilization of PR from crude membranes. Lane 1 (PR CM): PR in the crude membranes; lane 2 (PR-zSMALP1): PR solubilized into zSMALP1s. Equivalent volumes were loaded. (**D**) Solubilization of purified PR reconstituted into liposomes. Lane 1 (PR-PL): PR reconstituted into liposomes; lane 2 (PR-zSMALP1): PR solubilized into zSMALP1s. The equivalent volume run in lane 2 was twice that run on lane 1. The data correspond to immunoblots using a primary antibody against the His tag fused to the C-terminal end of PR.

We then tested for production of zSMALPs. The images in Fig. 4A show the solubilization of *E. coli* membranes containing proteorhodopsin (PR), which gives the pinkish color to the samples. The clear supernatant of the SMA-treated sample, after removal of insoluble material by centrifugation (tube 3), illustrates the known solubilizing power of SMA copolymers, which can directly dissolve cell membranes to produce SMALPs^12–14, 17^. However, addition of 5 mM MgCl_2_ (tube 4), CaCl_2_ (tube 5) or reducing the pH from 7.5 to 5.0 (tube 6) results in aggregation of the SMALPs. Figure 4B–D also show that zSMA1 can also solubilize cell membranes directly, but as expected from its design without the carboxyl acid groups (Fig. 1A), the resultant zSMALP1s remain in solution in the presence of divalent cations or after lowering the pH to 5 (Fig. 4B). This is consistent with the nearly neutral zeta potential of zSMA1 in solution (−2.8 mV *vs*. −36.7 of SMA (Supplementary Fig. 3).

**Figure 5:**
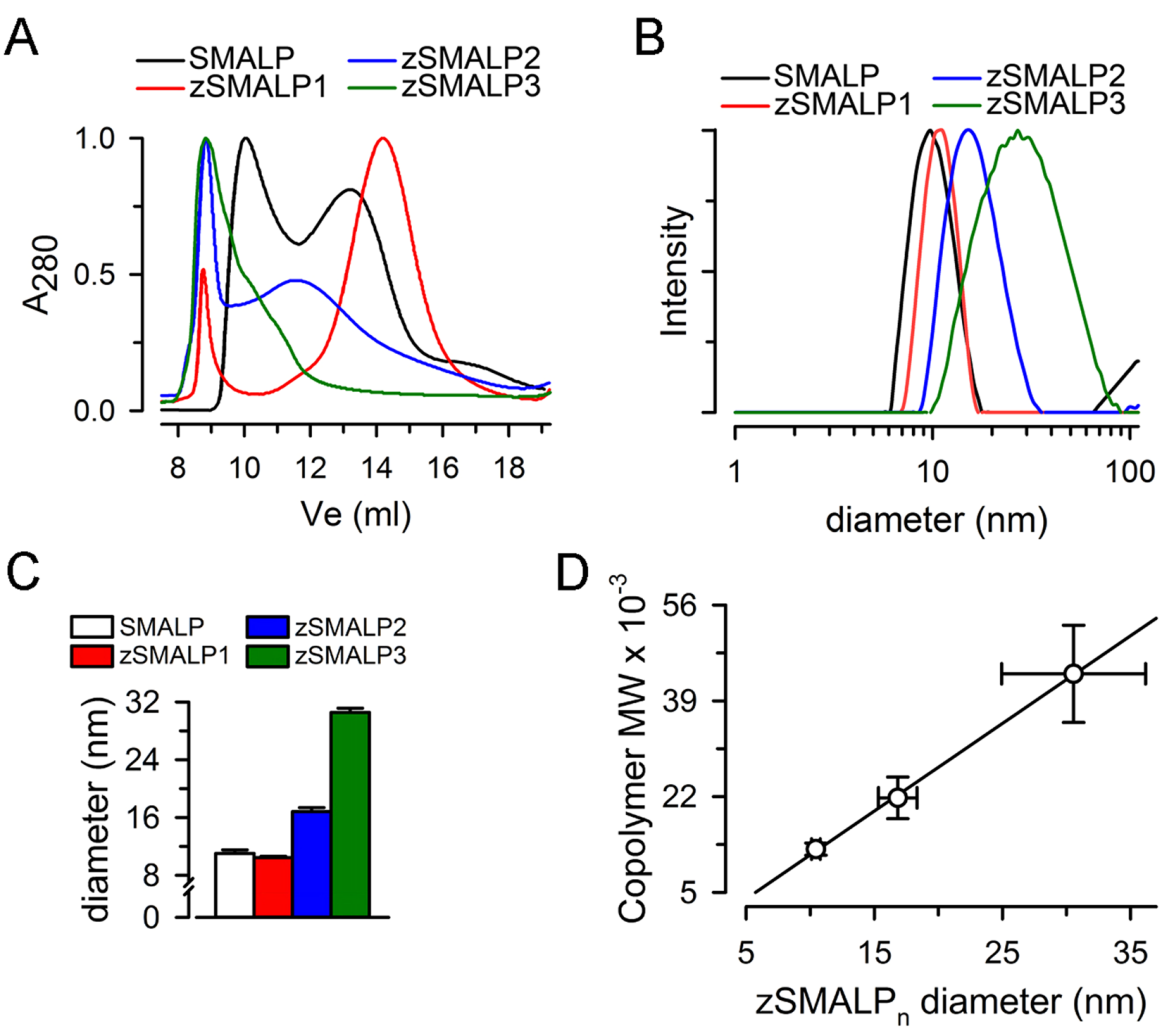
A comparison of nanodiscs formed by SMA and zSMAs of different sizes. (**A**) SEC. Liposomes formed by *E. coli* total lipids were solubilized with SMA, zSMA1, zSMA2 or zSMA3, and the solubilized material was run on a Superdex 200 Increase 10/300 GL size-exclusion column (see Methods for details). A_280_: absorbance at 280 nm. (**B**) Hydrodynamic diameter distribution. The main graph shows typical examples of different preparations illustrating the nanoparticle size distribution determined by dynamic light scattering. (**C**) Summary of hydrodynamic diameter data. Means ± SEM of experiments for nanodiscs formed by SMA (n = 4), zSMA1 (n = 6), zSMA2 (n = 5) and zSMA3 (n = 6). (**D**) Relationship between the molecular weight of the P(S-*at*-MA) alternating copolymers (determined by NMR, see Supplementary Table 2) and the zSMALPs diameter (determined by DLS, see panel B). Values are presented as means ± polydispersity. The line corresponds to a linear fit.

Solubilization of PR from *E. coli* crude membranes by zSMA1 was 62 ± 4% (n = 4), which is similar to the 55–60% reported for SMA copolymers^19, 22^. The size-exclusion chromatogram in Fig. 5A shows that the zSMALP1s formed by solubilization of model liposomes are more uniform in size than SMALPs, and that the size of zSMALP3 > zSMALP2 > zSMALP1; note that in our column zSMALP3s show in a shoulder that runs close to the aggregates. The hydrodynamic diameters for the peaks were determined by dynamic light scattering (DLS) and are shown in Fig. 5B. The values for SMALPs are consistent with previous estimations^12^. As expected from the data in Fig. 5A, the size of zSMALP1s is similar, but slightly more uniform than that of SMALPs (Fig. 5B), likely due to the narrower zSMA1 molecular weight distribution as a result of controlled/“living” free radical polymerization (Fig. 2). The DLS polydispersity index (PDI) calculated for zSMALP1s (0.033 ± 0.007; n = 6) was smaller than that for SMALPs (0.101 ± 0.022; n = 4; P < 0.01). The averages in Fig. 5B show that the diameters correlate with the size of the copolymer (zSMA3 > zSMA2 > zSMA1 ≅ SMA), suggesting the possibility of controlling the size of zSMALPs for specific applications by polymer engineering. This is further illustrated by the linear relationship between the copolymer molecular weight and the diameter of the zSMALPs (Fig. 5D). Figure 5D also shows that the PDI increased with the size of the copolymers, which may partly reflect the wider molecular weight distribution of zSMAs when their sizes get larger (Supplementary Table 2).

**Figure 6:**
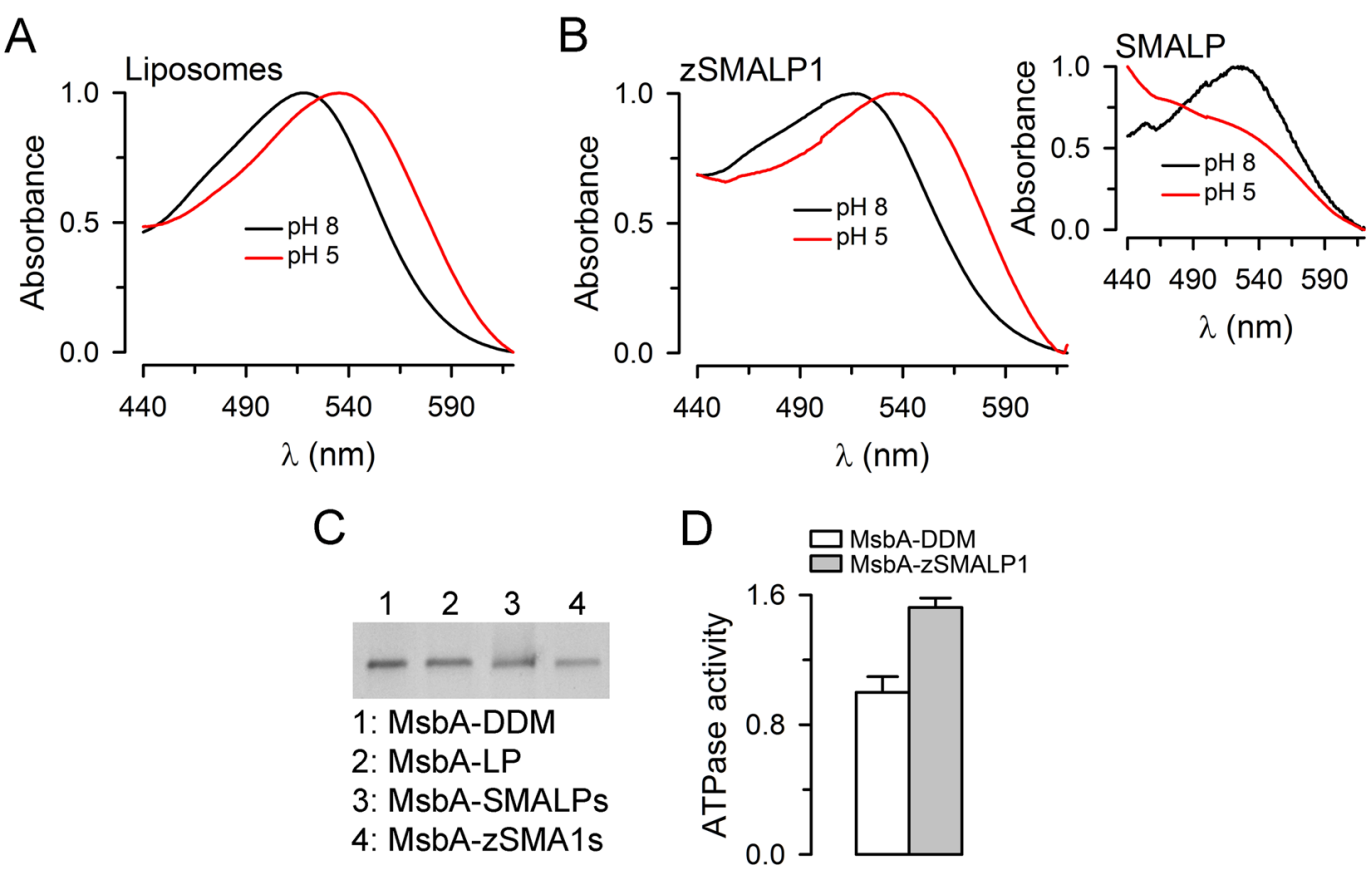
Activity assays of PR and MsbA in nanodiscs formed by zSMALP1. (**A**) Spectral shift of PR reconstituted in liposomes in response to lowering pH from 8 to 5. The data were normalized to the peak absorbance. (**B**) Spectral shift of PR reconstituted in zSMALP1s in response to lowering pH from 8 to 5. The data were normalized to the peak absorbance. The inset shows that the emission maximum is not clearly identifiable at pH 5 with the PR in SMALPs. At pH 5 the A_520_ of PR in SMALPs decreased to 39% of the value at pH 8 due to precipitation (n = 2; see Fig. 1A, tube 6). In contrast, the A_520_ of PR in zSMALP1s at pH 5 did not decrease (138 ± 13%; n = 3). (**C**) Solubilization/reconstitution of MsbA into SMALPs and zSMALP1s. Lane 1 (MsbA-DDM): purified MsbA in DDM; lane 2 (MsbA-LP): purified MsbA reconstituted into liposomes; lane 3 (MsbA-SMALP): MsbA reconstituted into liposomes and solubilized into SMALPs; lane 4 (MsbA-zSMALP1): MsbA reconstituted into liposomes and solubilized into zSMALP1s. The data correspond to bands stained with Instant Blue (Expedeon). (**D**) ATPase activity of MsbA. The ATPase activity of MsbA in zSMALP1s was slightly higher than that MsbA in the detergent DDM (P < 0.005; n = 6 for each condition). The data were normalized to the average activity in DDM (0.34 ± 0.03 ATP/s; n = 6). The activity of MsbA in SMALPs could not be measured because of the precipitation of MsbA-SMALPs in the presence of MgCl_2_.

We then focused on testing the usefulness of zSMA1 for functional studies of MPs that require divalent cations or low pH. The spectral shift elicited by lowering pH is a basic activity assay of rhodopsin-like proteins such as PR. For these studies, purified PR incorporated in model liposomes was solubilized with SMA or zSMA1 (see Methods and Fig. 4). The typical spectral shift produced by lowering pH (Fig. 6A)^23^ can be easily seen in the PR-loaded zSMALP1s, whereas such an assay cannot be performed in SMALPs because of the precipitation upon lowering pH (Fig. 6B and Fig. 6B inset). The shift in wavelength maxima by lowering pH from 8 to 5 was similar for PR in zSMALP1s (19.8 ± 0.2 nm; n = 3) and PR in detergent or liposomes (18.0 ± 0.8 nm; n = 4). Similarly, purified MsbA (an ATP-binding cassette transport ATPase) reconstituted in liposomes can be solubilized into SMALPs and zSMALP1 (Fig. 6C). However, the ATPase activity of this ABC protein can be evaluated only with the protein reconstituted into zSMALP1 (Fig. 6D) because the Mg^2+^ required for its ATPase activity causes precipitation of SMALPs (Fig. 4).

## Discussion

SMALPs are highly desirable because SMA copolymers can solubilize membranes directly, producing NDs with a near-physiologic environment for MPs that includes the native lipids, and avoids using detergents^12, 14^. SMALPs can also be produced starting with synthetic membranes of well-controlled compositions for studies of the regulatory role of lipids on MPs^12–14^. One major drawback of SMALPs is that aggregation and precipitation precludes their use in the presence of millimolar concentrations of polyvalent cations or at low pH. Recently, a diisobutylene/maleic acid copolymer (DIBMA) was tested for solubilization and formation of nanoparticles equivalent to SMALPs^18^. This copolymer was shown not to precipitate in the presence of divalent cations^18^, but it will likely precipitate at acidic pH because it contains the same carboxyl acids as SMA^24^.

In this study we present data on the formation of zSMALPs by all three zSMA copolymers and focus on zSMA1 because it has an average molecular weight close to that of the Xiran SMA control sample. Our zSMA1 produces zSMALPs similar in size to SMALPs formed by the Xiran control and other SMA copolymers reported in the literature. We showed the successful development of a new family of SMA copolymers in which we replaced the anionic carboxyl acid groups of conventional SMAs with zwitterionic PC groups. These new zSMAs can solubilize MPs directly from cell membranes or specifically formulated proteoliposomes to produce zSMALPs. Different from SMALPs, the new zSMALPs are compatible with buffers at low pH and in the presence of multivalent cations at millimolar concentrations. Our results also show that it is possible to control the size of the zSMALPs by tuning the zSMA copolymer sizes.

The new developments presented here will expand the use of polymer-encased bilayer nanostructures to studies of a large group of MPs in their functional states, including P-type, F-type, V-type and ABC ATPases, and allow for studies of the regulation of MPs by pH and divalent cations.

## Methods

### Materials

All chemicals employed were of the highest quality/purity available, and were used as received, except for styrene (inhibited with ~0.005% 4-tert-butylcatechol), AIBN and DATC. Styrene (Sigma-Aldrich) was purified by passing it through a basic aluminum oxide (Al_2_O_3_) column before use, and AIBN (Sigma-Aldrich) was re-crystallized from methanol twice before use. DATC was synthesized according to literature^25^. The commercially available SMA control sample (Xiran SL25010-S25) was a gift from Polyscope Polymers (Geleen, The Netherlands).

### Synthesis of cysteamine-PC modified alternating polymer P(S-*at*-MA)

In a typical reaction (Fig. 1B, scheme 1), styrene (2.08 g, 20 mmol), maleic anhydride (2.00 g, 20.4 mmol), DATC (18.3 mg, 0.05 mmol) and AIBN (1.6 mg, 0.01 mmol) were dissolved in 5.6 ml tetrahydrofuran (THF) in a 10-ml Schlenk flask, stirred, and degassed by three freeze-pump-thaw cycles. Then, the flask was sealed and immersed in a 70 °C-oil bath, and after a predetermined time the reaction was quenched by liquid nitrogen. The mixture was then diluted with THF and precipitated three times with an excess of ether/chloroform. The reaction conditions and conversions are summarized in Supplementary Table 1. To synthesize cysteamine modified phosphatidylcholine, 2-methacryloyloxyethyl phosphorylcholine was reacted with cysteamine *via* the thiol-ene “click” reaction^26^ (Fig. 1B, scheme 2). Briefly, cysteamine (0.74 g, 9.50 mmol), 2-methacryloyloxyethyl phosphorychroline (2.80 g, 9.48 mmol) and DMPP (66.3 mg, 0.48 mmol) were dissolved in 8 ml of dimethylsulfoxide (DMSO) in a 25-ml flask. After stirring for 2 days at room temperature the solution was precipitated twice with acetone/ether (2/1). The total amount of product (cysteamine-PC) was 3.3 g, with a yield of 93.2%. P(S-*at*-MA) was modified with the cysteamine-PC as shown in Fig. 1B (scheme 3). In a typical run, P(S-*at*-MA) (0.12 g, 0.6 mmol maleic anhydride) dissolved in 10 ml of DMSO was added into a 50-ml flask containing cysteamine-PC (0.60 g, 1.61 mmol), dicyclohexylcarbodiimide (DCC) (0.31 g, 1.5 mmol) and 15 ml of DMSO. After stirring for 2 days at room temperature, 5 ml of water was added to the mixture following by stirring for 30 min. The undissolved solid formed was filtered away and the solution was dialyzed in water for two days. The solvent was evaporated after filtering away the insoluble material, and the product was re-dissolved with DMSO/H_2_O and then precipitated twice into acetone/ether (3/1). The product was collected by centrifugation, dried in vacuum and characterized by ^1^H NMR.

### Characterization and Data Analysis

The chemical structures of the SMA polymers were characterized by ^1^H NMR (Jeol 400 MHz liquid-state NMR spectrometer), and the polymer size distribution was assessed by SEC. SEC was performed on an Agilent 1260 HPLC system equipped with a Wyatt Optilab T-rEX refractive index detector and a Wyatt MiniDAWN TREOS multi-angle light scattering detector, using an Agilent PLgel 5 µm MIXED-D column (300 × 7.5 mm). The system was equilibrated with dimethylformamide (DMF)/0.02 M ammonium acetate and run at 0.5 ml/min and 50 °C. The copolymerization of styrene and maleic anhydride is known to yield alternating polymers that contains a styrene/maleic anhydride repeating unit ratio close to 1:1 at equimolar feeding ratio of the two monomers^27^. We used NMR to characterize the ratio of styrene to maleic anhydride in the polymers. The sizes of the polymers were characterized by conversion analysis, NMR, and SEC. The chain transfer agent DATC has 16 protons in its hydrocarbon tail at 1.17–1.32 ppm (Supplementary Fig. 1A). This sharp peak was used as reference to characterize the structure of P(S-*at*-MA) because it is relatively undisturbed from other peaks. The NMR spectrum of P(S-*at*-MA)_59_ is shown in Supplementary Fig. 1B. When the peak at 1.17–1.32 ppm was set at 16 protons, there were 295 protons from the benzene ring of the styrene (*N*_*a*_) located at 5.70–8.50 ppm. The chemical shift of protons from the polymer backbone was mixed with those from DATC and solvents such as hexanes, acetone, DMF used in the synthesis and purification. As a result, the number of protons (*N*) from the backbone (*N*_*b*_) can be only calculated by subtracting the protons of all solvents (*N*_*solvents*_) and DATC (*N*_*DATC*_) from the total number of protons (*N*_*tot*_) in this range: *N*_*b*_ = *N*_*tot*_ − *N*_*solvents*_ − *N*_*DATC*_. Given that N_DATC_ is 31 and *N*_*solvents*_ from acetone-d_6_ and hexanes was determined as 58.83 and 3.05, respectively, *N*_*b*_ = 303.63. Since each styrene and maleic anhydride repeating unit contributes 3 and 2 protons to the polymer backbone, respectively, the average degree of polymerization (DP) of styrene can be calculated as *DP*_*S*_ = *N*_*a*_/5 = 59, and the average DP of maleic anhydride (*DP*_*MA*_) as: *DP*_*MA*_ = (*N*_*b*_ − *DP*_*S*_ × 3)/2 = 63. The *DP*_*MA*_/*DP*_*S*_ ratio was hence calculated to be 1.07, which is very close to 1/1 and the feeding ratio of the two monomers. The same method was used to calculate the DP and repeating unit ratio of the other two P(S-*at*-MA) copolymers, which were identified as P(S-*at*-MA)_106_ (Supplementary Fig. 1C) with *DP*_*S*_ = 106 and *DP*_*MA*_/*DP*_*S*_ = 1.00, and P(S-*at*-MA)_215_ (Supplementary Fig. 1D) with *DP*_*S*_ = 215 and *DP*_*MA*_/*DP*_*S*_ = 1.00, respectively. The molecular weight calculated from NMR data agrees well with that obtained by conversion analysis and SEC (Supplementary Table 2).

Cysteamine-PC was also characterized by NMR (Supplementary Fig. 2A). The chemical shifts of 6 protons in the 2.50–2.80 ppm range corresponding to protons b, c and d shown on the structure of cysteamine-PC (Supplementary Fig. 2A inset) are used as reference. We determined accordingly ~4 protons at 4.10–4.45 ppm (h, i), ~2 protons at 3.80–4.10 ppm (g), 2 protons at 3.48–3.67 ppm (j), ~9 protons at 2.97–3.30ppm (k) and ~1 proton at 2.06–2.13 ppm (e). The values and chemical shifts of those protons are in agreement with the structure of cysteamine-PC, suggesting that our synthesis was successful.

NMR was also used to characterize the zSMA structures after we modified SMAs with cysteamine-PC. The styrene structure was not affected by the modification, and was used as reference to calculate the percentage of amide bond formation on individual maleic anhydride repeating units due to their reaction with cysteamine-PC. The NMR spectrum of cysteamine-PC modified P(S-*at*-MA)_59_ is shown in Supplementary Fig. 2B. The number of protons from the benzene located at 5.70–8.50 ppm (peak a) was set to 5. Since the ratio of maleic anhydride/styrene is nearly 1, when both carboxyl groups on each maleic anhydride unit were fully hydrolyzed with cysteamine-PC to form amide bonds, a total of 12 protons for peak g, h and f are expected. The reason to use these proton peaks to calculate the percentage of amide bond formation is that they are mostly free from overlapping peaks of other protons. From the NMR spectrum *N*_*g,h,f*_ = 9.84, and the percentage of amide bond formation was therefore ~82.0%. For cysteamine-PC modified P(S-*at*-MA)_106_ (zSMA2; Supplementary Fig. 2C) and P(S-*at*-MA)_215_ (zSMA3; Supplementary Fig. 2D), the percentage of amide bond formation was estimated similarly as 82.2% and 88.8%, respectively. We could not determine quantitatively to what extent the incomplete amide modification of the maleic anhydride repeating units was caused by: (1) unreacted carboxyl groups that exist as carboxyl acids; and (2) part of the modification proceeded to form imide instead of amide, respectively. Considering that the zeta potential of the zSMA1 solution is nearly neutral (Supplementary Fig. 3) and that none of the zSMAs precipitate at low pH or in the presence of polyvalent cations (Fig. 3), we speculate that the incomplete amide modification is most likely caused by imide formation, and that the presence of residue carboxyl acids on zSMAs is negligible.

### Zeta potential

For determinations of the zeta potential, the Xiran control and zSMA1 were dissolved to ~1 mg/ml in 100 mM NaCl, 50 mM Tris/HCl, pH 9.0, and the solutions were filtered through a 0.2-μm syringe filter before analysis. Determinations were performed using a Zetasizer Nano ZSP (Malvern Instruments).

### Expression and purification of proteorhodopsin

A synthetic gene (Genscript) coding for proteorhodopsin (PR) with a 6-His tag fused to the C-terminal end was cloned into the *NcoI*/*BamHI* sites of the expression vector pET19b. PR was overexpressed in the *E. coli* strain BL21 (DE3) codon plus (Agilent Technologies) transformed with the pET19-PR plasmid. The cells were grown at 37 °C in 2YT medium with 0.5% glucose, 200 μg/ml carbenicillin, 34 μg/ml chloramphenicol and 10 μM all-*trans* retinal. The cells were induced at OD_600_ ~1 with 1 mM isopropyl-β-D-thiogalactopyranoside (IPTG). At induction, we also added an amount of all-*trans* retinal equal to that present in the original medium, and the cells were harvested 4 h later. All subsequent procedures were performed at 4 °C unless specified otherwise. Cell pellets were resuspended in a buffer containing 50 mM potassium phosphate and 5 mM MgCl_2_, pH 7.2, with 10 μg/ml lysozyme, 10 μg/ml DNAse I, 1 mM phenylmethanesulfonyl fluoride (PMSF) and a protease inhibitor cocktail (1 tablet/100 ml of buffer; complete EDTA-free, Roche), and lysed on a microfluidizer. Crude membranes were prepared by centrifugation at 135,000 g for 1.5 h, and were solubilized overnight, at 4 °C, in a buffer containing 150 mM KCl, 50 mM potassium phosphate, pH 8, 1 mM PMSF and 1.5% n-dodecyl-β-D-maltopyranoside (DDM; Inalco Pharmaceuticals), at a total protein concentration < 2 mg/ml. The DDM-solubilized lysate was centrifuged at 100,000 g for 30 min, and the supernatant was incubated with Talon Co^2+^ beads (Talon Superflow, Clontech) for 3 h. The resin was washed with 10 column volumes of a buffer containing 150 mM KCl, 50 mM potassium phosphate, pH 8, 0.05% DDM and 40 mM imidazole. Imidazole was added to a concentration of 250 mM for elution. After elution, the imidazole was removed by SEC using a Bio-scale Mini Bio-gel P-6 desalting cartridge (Bio-Rad) equilibrated with 10 mM Tris/HCl, pH 8.2, with 0.05% DDM. The purity of the preparation was assessed by staining gels (16% SDS PAGE) with Instant Blue (Expedeon) and UV-Vis spectroscopy. The PR samples were concentrated to 10 mg/ml, and stored at 4 °C until use.

### Extraction of proteorhodopsin from crude membranes with SMA and zSMA1

For our comparative experiments we used the SMA copolymer Xiran SL25010-S25. As mentioned in the main text, we also refer to this copolymer as SMA. SL25010-S25 was provided by Polyscope Polymers in a sodium salt aqueous solution. Crude membranes from *E. coli* cells expressing PR were prepared as described above. The membranes were resuspended at a final concentration of 40 mg/ml (wet membrane mass) in 150 mM NaCl, 50 mM Tris/HCl, pH 8, and 10% glycerol. SMA or zSMA1 were added to a final concentration of 2.5% (w/v) and the samples were incubated at room temperature with gentle rotation for 2 h. Insoluble material was removed by centrifugation at 100,000 g for 30 min, at 4 °C, and the supernatant containing PR-loaded SMALPs or zSMALP1s was analyzed for buffer compatibilities by addition of MgCl_2_ or CaCl_2_ to a final concentration of 5 mM, or by lowering the pH from 8 to 5 with HCl. The presence of PR in the crude membranes and copolymer-solubilized samples was determined by Western blotting using an antibody against the 6x His tag (anti-Hexa-His antibody, GenScript) fused to the C-terminal end of PR. The secondary antibody was a goat-anti-mouse Alexa Fluor 680 (Life Technologies), and the signal was visualized using an Odyssey Infrared Imager (Li-Cor Biosciences). The efficiency of PR solubilization in zSMALPs was quantified from the Western blots using densitometry (UN-SCAN-IT, Silk Scientific).

### Solubilization/reconstitution of proteorhodopsin in SMALPs or zSMALPs

Stocks of 1,2-dioleoyl-*sn*-glycero-3-phosphocholine (DOPC), 1,2-dioleoyl-*sn*-glycero-3-phospho-(1′-*rac*-glycerol) (DOPG) in chloroform (Avanti Polar Lipids) were dried under a stream of argon followed by vacuum drying overnight. The dried lipids resuspended at a concentration of 10 mg/ml in 50 mM Tris/HCl, pH 8, with 4 mM DDM, were sonicated until clear. Proteoliposomes were obtained by reconstituting DDM-solubilized PR at 2:1 protein:lipid ratio (w/w) by SEC (Zeba columns, Thermo Fisher Scientific) pre-equilibrated with a solution containing 100 mM NaCl and 50 mM Tris/HCl, pH 8^28, 29^. The lipids consisted of a 9:1 ratio (w/w) of DOPC:DOPG. After reconstitution, the samples were extruded through a 200-nm polycarbonate filter (Mini-Extruder, Avanti Polar Lipids). PR-liposomes were incubated with SMA or zSMA1 at a final concentration of 2.5% (w/v) for 2 h at room temperature. After incubation, non-solubilized material was removed by ultracentrifugation at 100,000 g for 30 min. The purity of the preparation was assessed in Coomassie blue-stained gels and by UV-Vis spectroscopy.

### Proteorhodopsin activity assay

The basic activity of PR was determined by the shift in absorption spectra elicited by lowering pH from 8 to 5. The absorption spectra of PR-loaded SMALPs or zSMALP1s, PR in DDM or liposomes were collected on a Jasco spectrophotometer (model V-630) at 22 °C. The spectra were recorded first at pH 8, and then after acidification to pH ~5 by addition of 1 N HCl.

### Preparation of liposomes and solubilization by SMA or zSMA copolymers

*E. coli* total lipids dissolved in chloroform (Avanti Polar Lipids) dried as described above were resuspended to a final concentration of 20 mg/ml in 100 mM NaCl, 20 mM Tris/HCl, pH 7.4, with 4 mM DDM, and sonicated until clear. Liposomes produced by SEC using Zeba spin columns pre-equilibrated with 100 mM NaCl and 20 mM Tris/HCl, pH 7.4, were extruded as described above. For solubilization and formation of SMALPs and zSMALPs the liposomes were incubated for 2 h at room temperature with SMA, zSMA1, zSMA2 or zSMA3 at a final concentration of 2.5% (w/v). The samples were centrifuged at 125,000 g for 30 min and the supernatants (200 μl) were analyzed by high-resolution SEC using a Superdex 200 Increase 10/300 GL column (GE Healthcare) equilibrated with the same buffer. The flow rate was set at 0.5 ml/min and fractions of 1 ml were collected for isolation of relevant peaks used for further studies.

### Estimation of SMALPs and zSMALPs size by dynamic light scattering

Dynamic Light Scattering (DLS) experiments were performed at 22 °C on a Zetasizer Nano ZSP (Malvern Instruments), using 40-μl disposable microcuvettes. For each determination, measurement were repeated at least 3 times, with each being a 15-scan average (each ~15-s long). Size-intensity distributions were generated using the Zetasizer software version 7.11.

### Solubilization/reconstitution and function of MsbA in SMALPs and zSMALP1s

MsbA was expressed, purified and reconstituted in liposomes as described^6, 30^. For reconstitution, purified MsbA in 0.065% DDM and 0.04% Na cholate was mixed at 1:10 protein:lipid ratio (w/w) with *E. coli* total lipids (Avanti Polar Lipids) in 100 mM NaCl, 20 mM Tris/HCl, pH 7.4, with 0.1 mM tris(2-carboxyethyl)phosphine (TCEP). The liposomes containing MsbA were extruded through a 200-nm polycarbonate filter and incubated with SMA or zSMA1 at a final concentration of 2.5% (w/v) for 2 h at room temperature. After incubation, non-solubilized material was removed by centrifugation at 100,000 g for 30 min. The MsbA-loaded nanodiscs were enriched based on the affinity of the His-tagged MsbA for Ni^2+^. The supernatant was mixed with Ni-NTA beads (Thermo Fisher Scientific) at a ratio of 100 μl of resin/ml of solubilized protein, and incubated at 4 °C overnight with gentle rotation. Then, the samples were transferred to a gravity flow column and the resin was washed with 10 column volumes of 100 mM NaCl, 20 mM Tris/HCl, pH 7.4, with 0.1 mM TCEP and 20 mM imidazole, and elution was achieved by increasing the concentration of imidazole to 200 mM. Eluted fractions were analyzed on gels (16% SDS-PAGE) stained with Instant Blue (Expedeon). The ATPase activity of MsbA was measured using a variant of the ATPase linked assay^30, 31^.

### Statistics

Statistical comparisons were performed by the Student’s *t* test for paired or unpaired data, or one-way analysis of variance, as appropriate. P < 0.05 in a two-tailed analysis was considered significant. The number of experiments given in the main text and figure legends corresponds to independent measurements.

### Data Availability

The datasets generated during and/or analyzed during the current study are available from the corresponding authors on reasonable request.

## Electronic supplementary material

Supplementary Information
