# Supplementary material for: Polymer-encased nanodiscs with improved buffer compatibility
Source: Sci Rep. 2017 Aug 7;7:7432. doi: 10.1038/s41598-017-07110-1 (PMC5547149; doi:10.1038/s41598-017-07110-1)
Supplement: Supplementary file 1 — Supplementary Information [file 41598_2017_7110_MOESM1_ESM.pdf]

SUPPLEMENTARY INFORMATION

Polymer-encased nanodiscs with improved buffer compatibility

Mariana C Fiori, Yunjiang Jiang, Guillermo A Altenberg & Hongjun Liang

Department of Cell Physiology and Molecular Biophysics, and Center for Membrane Protein Research, Texas Tech University Health Sciences Center, Lubbock, TX, USA.

SUPPLEMENTARY TABLES

Supplementary Table 1. Conditions for the RAFT polymerization of the P(S-*at*-MA) copolymers and conversion.

Sample	[Monomer]	[S]/[MA]/[CTA]/[I]	Reaction time	Conversion
P(S-*at*-MA)$_{59}$	5 M	59/62/1/0.125	6 h	99%
P(S-*at*-MA)$_{106}$	5 M	400/408/1/0.125	2 h	26%
P(S-*at*-MA)$_{215}$	5 M	400/410/1/0.125	3.5 h	53%

S: styrene; MA: maleic anhydride; CTA: cysteamine; I: initiator, azobisisobutyronitrile (AIBN).

Supplementary Table 2. Molecular weights and polydispersity indexes before PC modification.

Sample	MW from conversion	MW from NMR	GPC in DMF	
			Mn	PDI
P(S-*at*-MA)$_{59}$	12,451	12,675	NA	1.085
P(S-*at*-MA)$_{106}$	21,576	21,777	35,000	1.170
P(S-*at*-MA)$_{215}$	43,708	43,795	53,800	1.197
SMA (Xiran)	NA	NA	NA	1.341

MW: molecular weight; GPC: gel permeation chromatography; DMF: dimetylformamide; Mn: number average molecular weight; PDI: polydispersity index; NA: not available.

SUPPLEMENTARY FIGURES

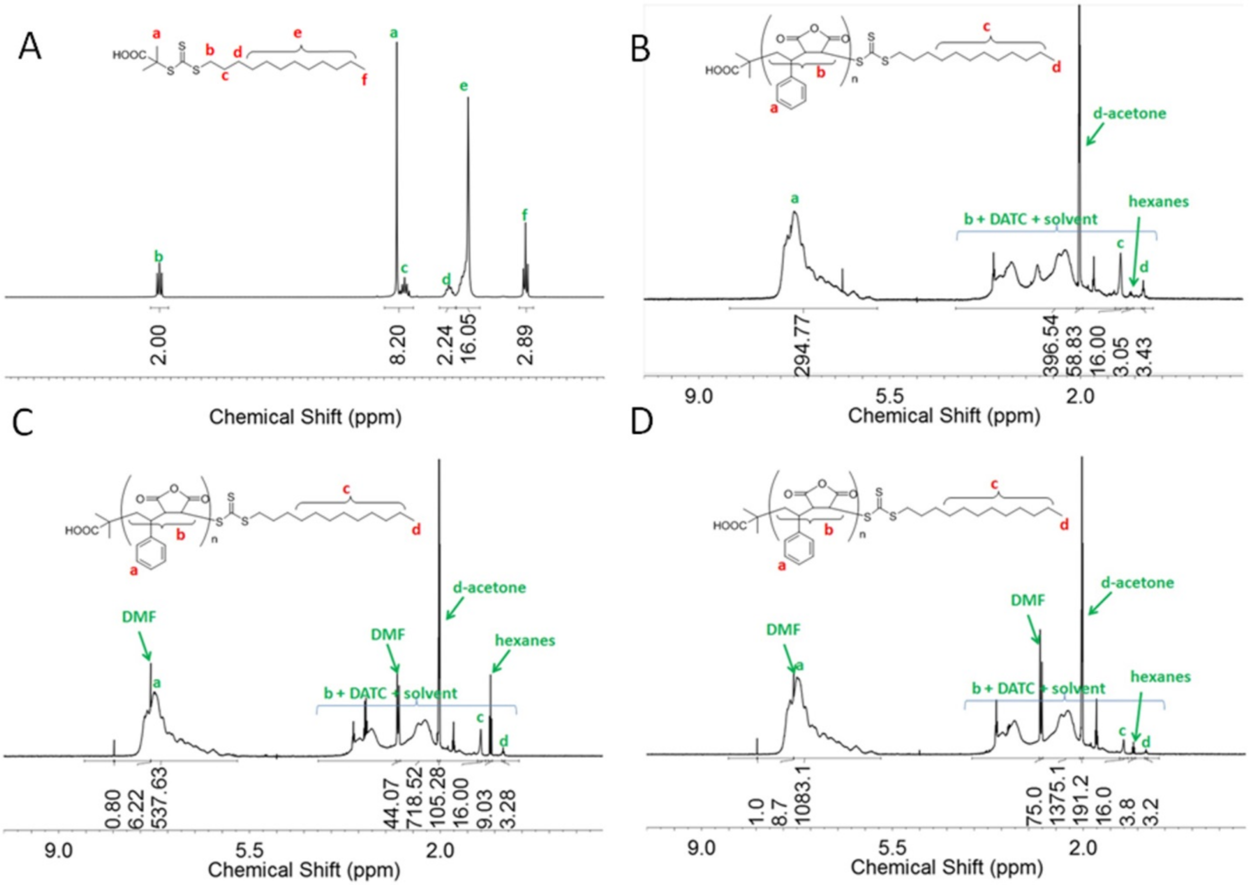

Supplementary Figure 1. NMR spectra for the synthesis of P(S-*at*-MA) alternating copolymers. (A) NMR spectrum of DATC. (B) NMR spectrum P(S-*at*-MA)$_{59}$. (C) NMR spectrum of P(S-*at*-MA)$_{106}$. (D) NMR spectrum of P(S-*at*-MA)$_{215}$. DATC was dissolved in $CHCl_3$ and the alternating copolymers were dissolved in acetone-d_6.

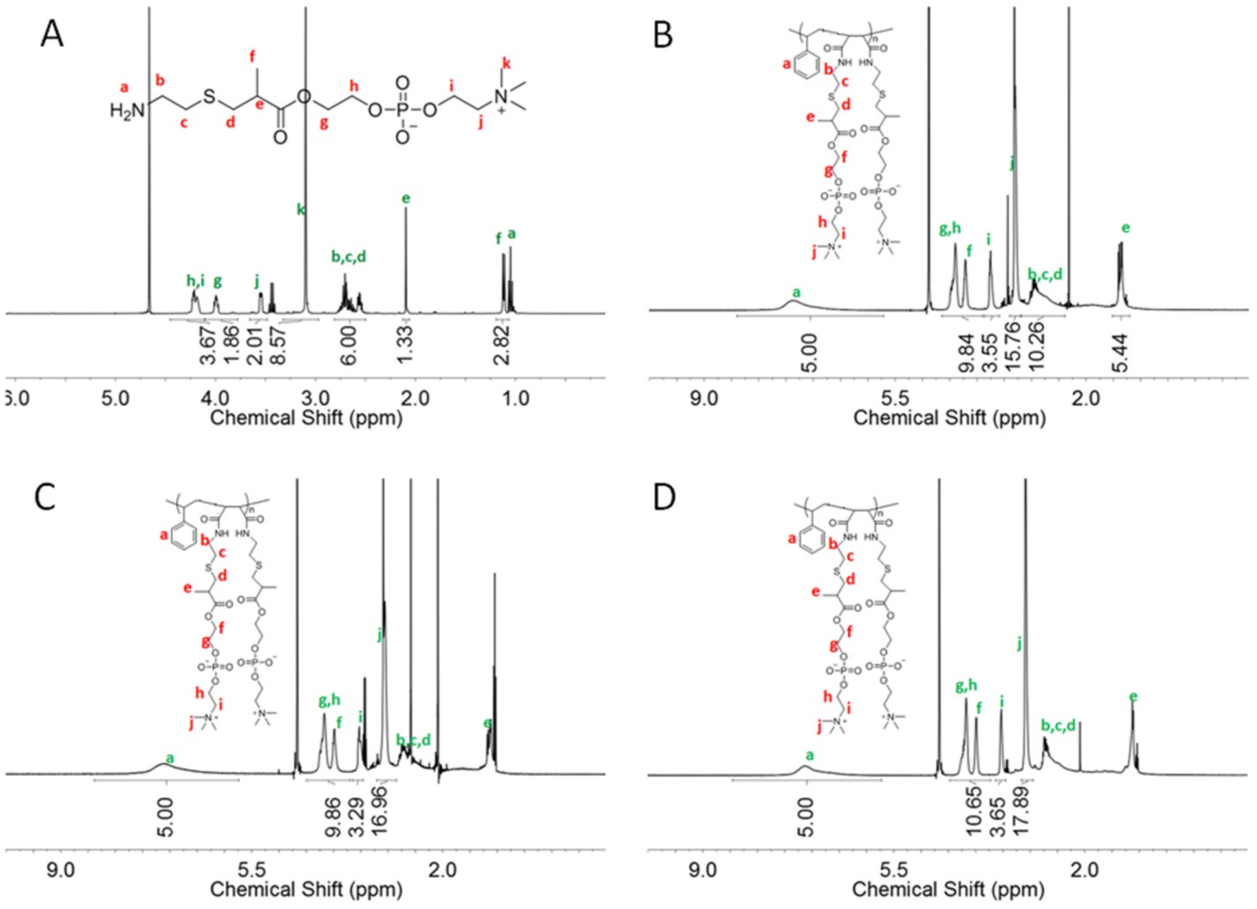

Supplementary Figure 2. NMR spectra for the synthesis of zSMAs. (A) NMR spectrum of cysteamine-PC. (B) NMR spectrum of zSMA1. (C) NMR spectrum of zSMA2. (D) NMR spectrum of zSMA3. All compounds were dissolved in D_2O.

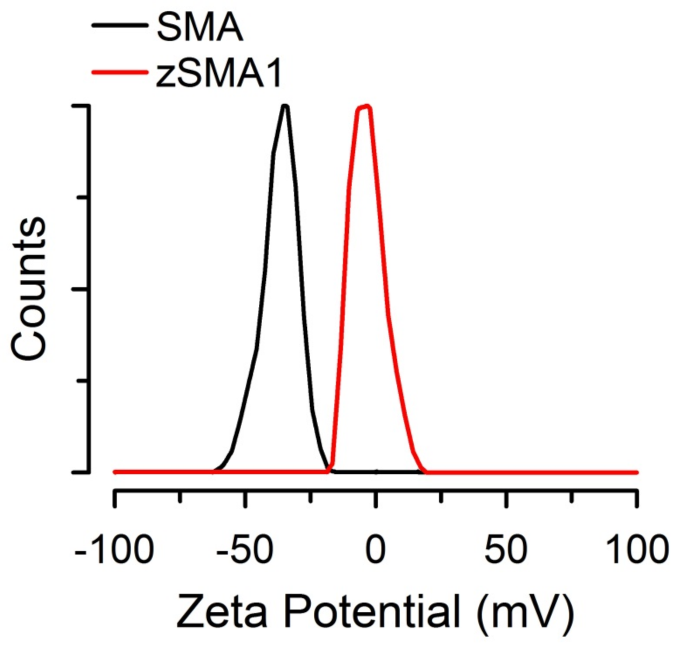

Supplementary Fig. 3. A comparison of zeta potentials. Apparent zeta potential of the Xiran SMA control trace and zSMA1 measured in 100 mM NaCl and 50 mM Tris/HCl, pH 9.0. The average zeta potential of commercial SMA and zSMA were -36.7 and -2.8 mV, respectively.
